# Evaluation of image processing technique in identifying rice blast disease in field conditions based on KNN algorithm improvement by K‐means

**DOI:** 10.1002/fsn3.1251

**Published:** 2019-11-07

**Authors:** Mohammad Reza Larijani, Ezzatollah Askari Asli‐Ardeh, Ehsan Kozegar, Reyhaneh Loni

**Affiliations:** ^1^ Department of Biosystems Engineering University of Mohaghegh Ardabili Ardabil Iran; ^2^ Department of Computer Sciences and Engineering University of Guilan Guilan Iran; ^3^ Department of Biosystem Engineering Tarbiat Modares University Tehran Iran

**Keywords:** blast disease, image processing, K‐means algorithm, KNN algorithm, rice

## Abstract

Nowadays, rice farming is affected by various diseases that are economically significant and worthy of attention. One of these diseases is blast. Rice blast is one of the most important limiting factors in rice yield. The purpose of this study is the timely and rapid diagnosis of rice blast based on the image processing technique in field conditions. To do so, color images were prepared using image processing technique and improved KNN algorithm by K‐means was used to classify the images in *Lab* color space to detect disease spots on rice leaves. Squared classification was based on Euclidean distance, and the Otsu method was used to perform an automatic threshold histogram of images based on shape or to reduce the gray level in binary images. Finally, to determine the efficiency of the designed algorithm, sensitivity, specificity, and overall accuracy were examined. The classification results showed that the sensitivity and specificity of the designed algorithm were 92% and 91.7%, respectively, in the determination of the number of disease spots, and 96% and 95.65% in determining the quality of disease spots. The overall accuracy of the designed algorithm was 94%. Generally, the results obtained showed that the above method has a great potential for timely diagnosis of rice blast.

## Graphical abstract

Rice blast caused by fungus Magnaporthe oryzae is generally considered the most important disease of rice worldwide because of its extensive distribution and destructiveness under favorable conditions. In case of severe disease, all leaves of a plant may become dry. There are chemical methods to prevent this fungal disease, but the important point is the timely and accurate diagnosis of the disease. Considering the significance of the topic, it is very important to use the science of machine vision and image processing techniques, which today play a major role in precision agriculture.

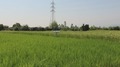

## 1 INTRODUCTION

In scientific terms, rice is called *Oryza Sativa*. It is one of the most important and strategic crops and it is the food of more than two third of the population of the world. Rice is considered as a healthy and nutritious foodstuff, and now it provides 50% of the world's agricultural production and 20% of the energy needed by humans. The study of rice from the viewpoint of food security shows that over the past three decades, the use of rice in Iranian people's eating habits has been increasing steadily, with per capita consumption ranging from 15–20 kg, 30–40 years ago, to higher than 37.4 kg in the last decade (Peyman, Bakhshipour Ziaratgahi, & Jafari, 2016).

However, population growth and growing demand for this product have placed Iran among the most important countries importing rice. Therefore, focusing on the increase in production per unit area is the most important goal of the country in the field of agriculture in order to apply all the factors affecting production. But rice farming is endangered by many pests and diseases that are economically significant and need to be taken into consideration carefully (Agahi, Fotokian, & Younesi, 2012; Peyman et al., 2016). One of these rice diseases is blast. Blast is one of the most important limiting factors in plant performance. The maximum sensitivity to the blast disease is observed at the sprouting stage, that is when the rice plant starts to appear above the surface of the ground. Even resistant varieties are susceptible to this disease during the formation of flowering organs. Symptoms of the disease on the leaf appear first in the form of burned water points and then turn into rhombus‐shaped spots of 1–3 cm in length which are tipped at the end. The spots are gray in the center and dark brown in the margin. In case of severe disease, all leaves of a plant may become dry. There are chemical methods to prevent this fungal disease, but the important point is the timely and accurate diagnosis of the disease (Kumar et al., 2003). Because of the very high importance of blast disease, extensive research has been conducted to control it from various aspects (diagnosis, use of pesticides and fungicides, effective factors in reducing the disease, heat and moisture). The cause of the spread of this disease in the rice fields in northern Iran has been overuse of nitrogen fertilizers (Khosravi, 2006). So far, with the management of plant nutrition, planting time, rotation plans, plant spacing, soil moisture, biological control, and chemical control, no considerable results have been achieved in the fight against this disease (Ashkani et al., 2016; Bakar et al., 2018). Considering the significance of the topic, it is very important to use the science of machine vision and image processing techniques, which today play a major role in precision agriculture. The machine vision, as a powerful and reliable tool, has been widely used in various industries, especially in agriculture. The main use of machine vision and image processing in agriculture involves controlling the status of agricultural land, precision agriculture (PA), controlling and supervising plants at the planting stage and controlling and monitoring the quality of agricultural products at the postharvest stage. The main reason for the ever‐increasing expansion of the machine vision and image processing science in various branches of agricultural science is that in addition to identifying the shape, color, size and texture of the objects, these systems can also extract the numerical and quantitative characteristics of these objects (Azarmdel, Mohtasebi, Jafari, & Muñoz, 2019; Jahanbakhshi & Kheiralipour, 2019; Kahar, Mutalib, & Abdul‐Rahman, 2015; Pydipati, Burks, & Lee, 2006).

Nowadays, rapid development of computer processing technologies and creation of related software makes it possible for us to benefit from the advantages of artificial intelligence. One example of these technologies is the application of artificial neural networks and other algorithms that to some extent copy human brain functions, to solve problems in systems and modeling processes (Jahanbakhshi, Ghamari, & Heidarbeigi, 2017; Kaveh, Jahanbakhshi, Abbaspour‐Gilandeh, Taghinezhad, & Moghimi, 2018). Samanta & Ghosh (2012) used an artificial neural network to detect pests on tea tree shrubs applying the CFS‐based McCulloch‐Pitts model for designing a disease identification algorithm. The results of the experiments based on the designed algorithm had a 100% precision in pest detection. Pydipati et al. (2006) identified four grapefruit‐related diseases using image processing and isolation analysis techniques and applied the co‐occurrence matrix for classification. They were able to classify the diseases with the precision of about 98.75%. Al‐Saqer (2012) used artificial neural networks to detect tropical walnut mite pests. His test results showed with 100% accuracy that the detection algorithm can perform diagnostic operations. In addition, the pest analysis and detection time by the algorithm in question was 0.16 s. Kahar et al. (2015) used artificial neural network and fuzzy logic to categorize three types of rice disease, that is blast, rice pod rodent, and rice leaf rodent. The accuracy of their algorithm in the diagnosis of rice plant disease was 100%.

In modern agriculture, quick methods reviews, automated, cheap and accurate methods for diagnosing plant diseases are important. Timely and accurate diagnosis of disease in farms is one of the most important factors in controlling plant diseases. Also, the use of a method that can manage the whole farm to be online is very important. Therefore, the purpose of this research is to online management of rice fields using a quadcopter (helicopter) and image processing technique to identify rice blast disease in field conditions. Review of the related literature revealed that until now no studies have been conducted on detecting the pests and diseases of the rice plant to be online using a quadcopter and image processing technique.

## 2 MATERIALS AND METHODS

### 2.1 Field imaging and preparation of the images

**Figure 1:**
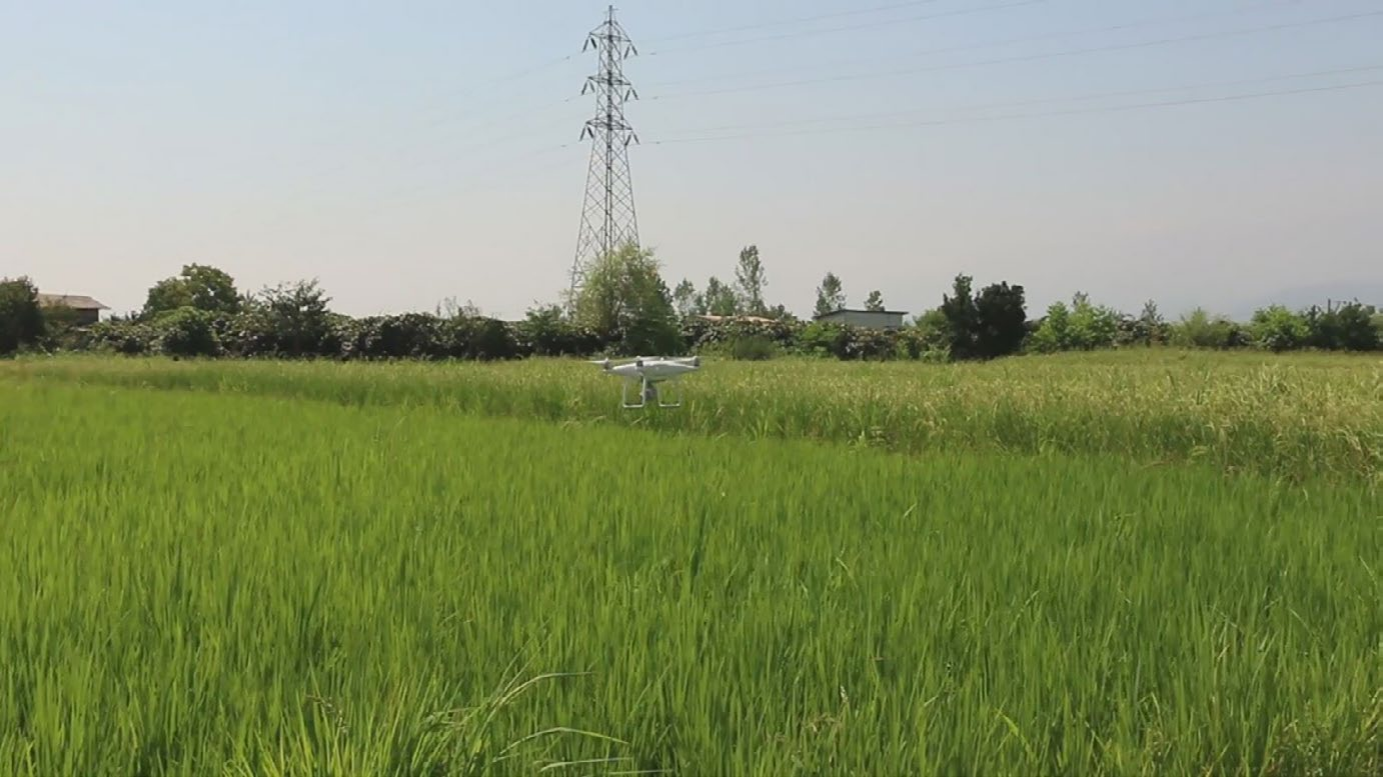
Imaging by quadcopter

Canopy color images of rice fields were prepared in RGB space by a PHANTOM 4 ADVANCED quadcopter, equipped with a 12‐megapixel digital camera. The velocity of the quadcopter in rice fields was 0.5 m/s and its distance from the bushes was 90 cm (Figure 1). The images were prepared under different weather conditions almost four weeks after planting rice. Due to the filming of different parts of the farms, the dates of planting the rice bushes were not the same.

**Figure 2:**
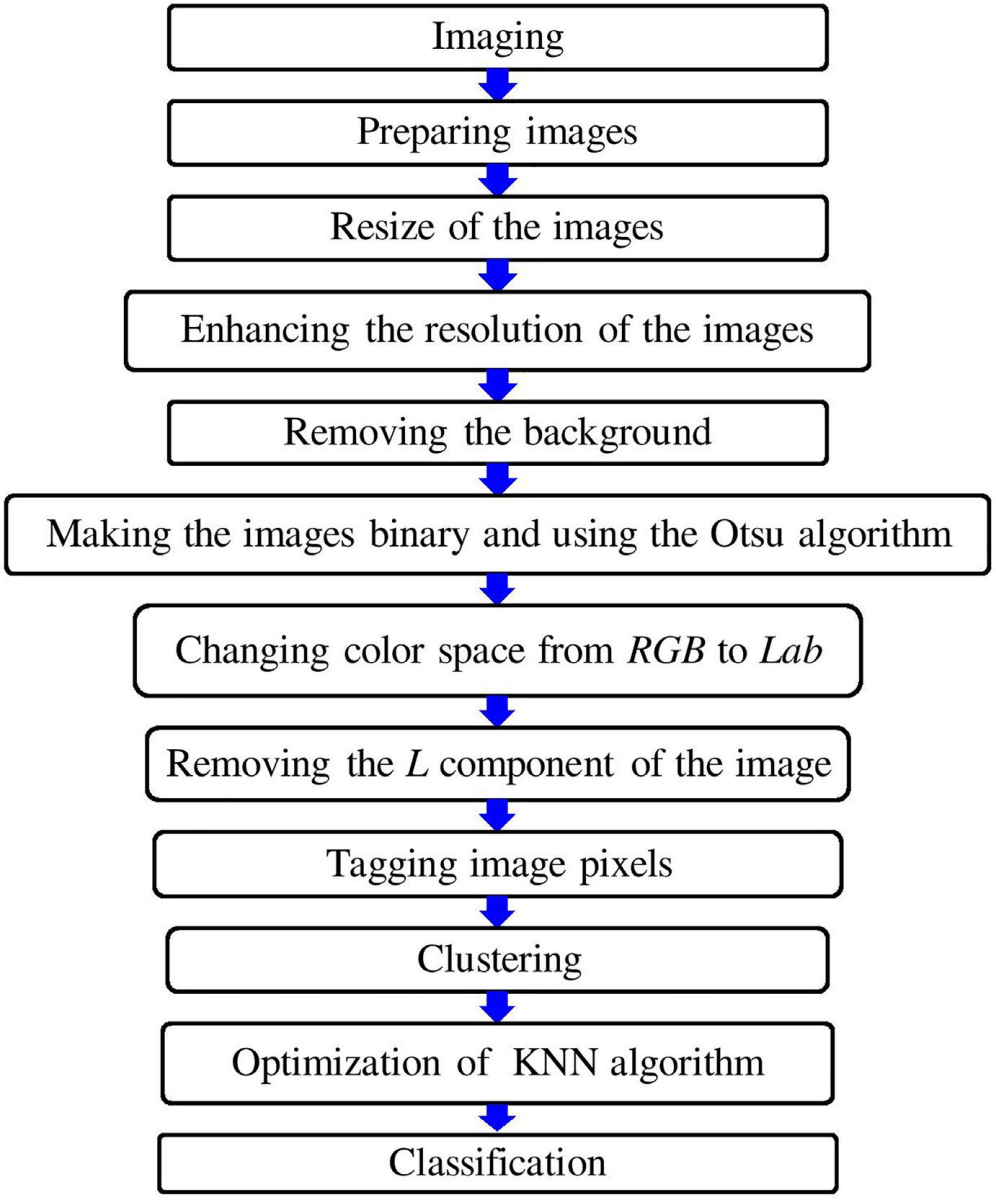
Stages of analysis of rice leaf images

The stages of analysis of rice leaf images for detecting blast disease under field conditions based on the K‐means and KNN algorithms are presented in Figure 2.

### 2.2 Preprocessing images

**Figure 3:**
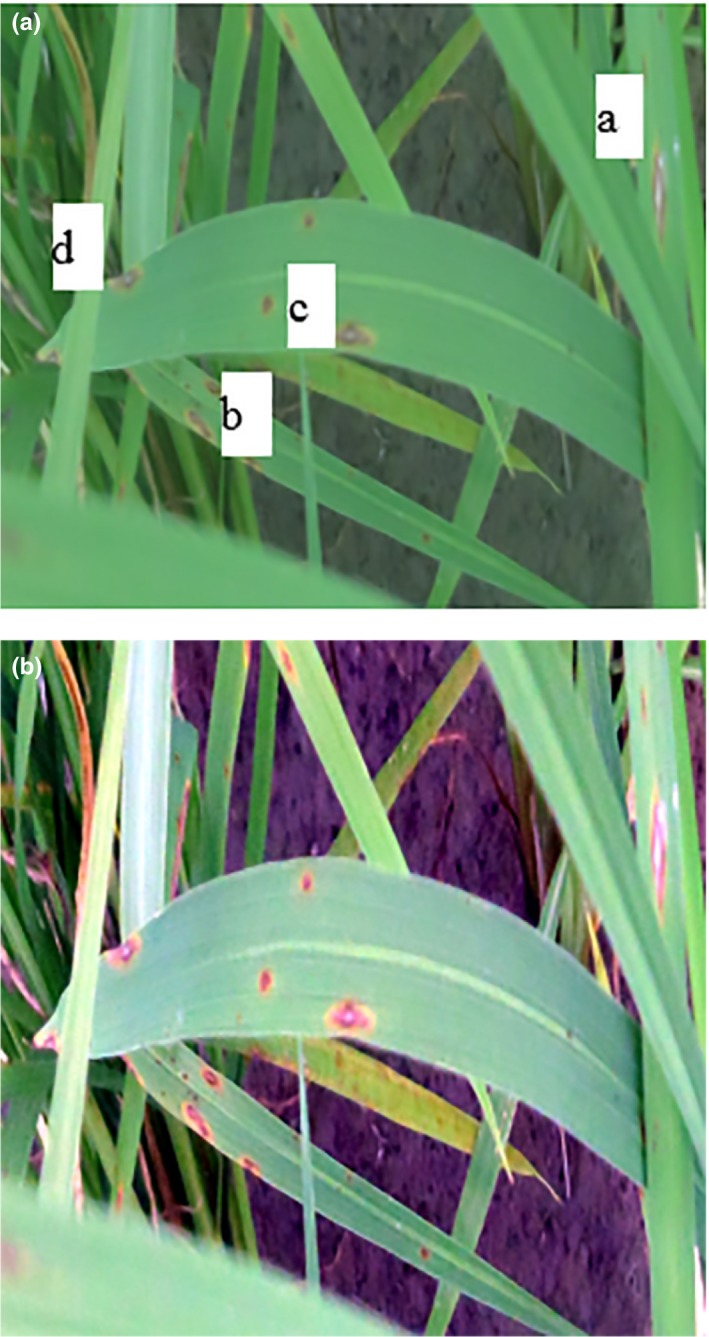
(a) Input image of the algorithm and (b) High‐resolution image

Image processing started after the imaging stage was completed. To process images, an application was coded in MATLAB R2012a. After the images were invoked by the program, the preprocessing of the images began. Because the images were large and this reduced the speed of the analysis and processing of images, the images were converted to 256 × 256 pixels so that the computing machine could perform image analysis in the shortest possible time. Improving the resolution of an image to better diagnose the disease and to raise the ability to diagnose the healthy surfaces from the diseased ones (points a, b, c and d in Figure 3). Therefore, the designed algorithm could better detect the disease on the plant leaf (Figure 3).

Because the images were taken at different times of the day and different weather conditions with different light intensities, the lighting and image alignment operations were then carried out in the next step. This allows the image to match with the image pixels.

### 2.3 Image processing

**Figure 4:**
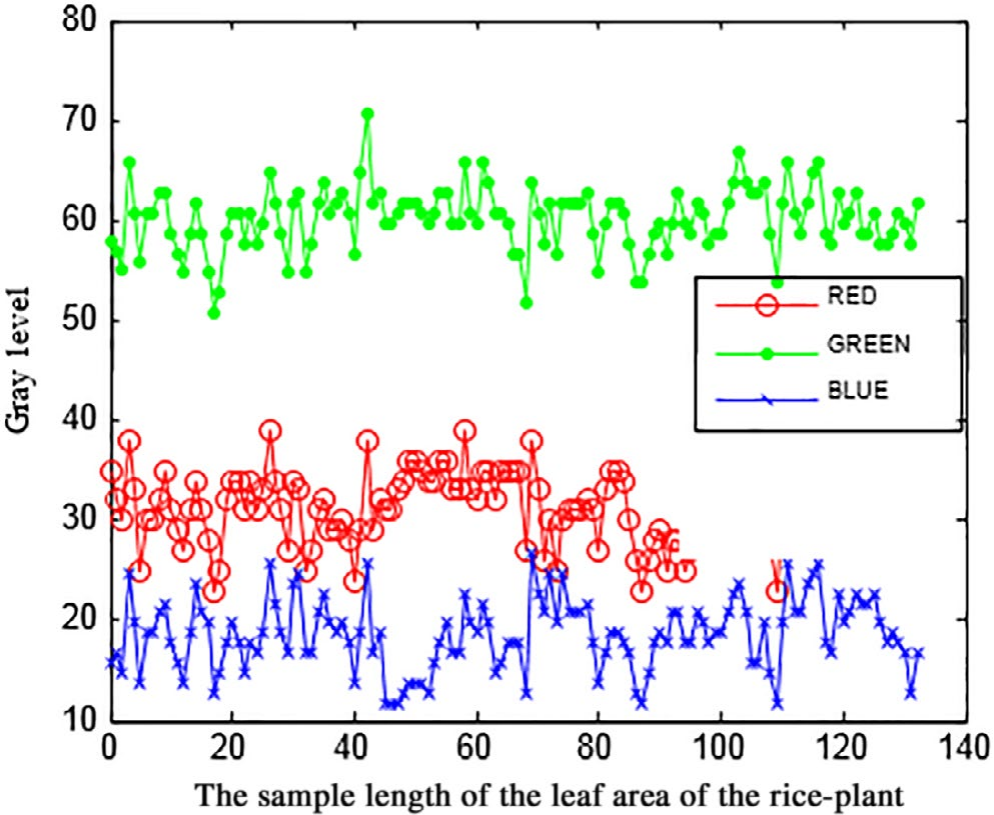
Profile extracted from the rice leaf

**Figure 5:**
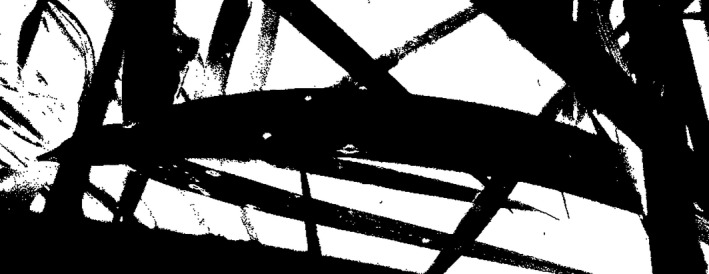
Removing the background image of the rice bush

In the next step, the separation algorithm was performed using the histogram profile extraction (Figure 4) to remove the probable background in the received images. The received images included rice bushes, stems, and green leaves and mud (predominantly green and gray colors). In this algorithm, the original image was first split into three components: red, green, and blue, and then the rice plant was removed from the background of the image (Figure 5).

### 2.4 Final processing

To perform segmentation, the received images must be transferred to the color space independent of the device. In a color space independent of the device, the coordinates used to determine the color determine the same color, regardless of the device used, and *a* color‐dependent device is the space in which the resulting color depends on the equipment used to produce it (Kubat, 2015). Therefore, in this study, *Lab* color space was used which is widely used in machine vision. The three dimensions of this color space are the color brightness (*L* = 0 indicates black color and *L* = 100 shows white), its position relative to red, purple or green (*a*: negative values indicate green and positive values indicate violet), and its position relative to the yellow and blue (*b*: the negative values indicate a blue and the positive values indicate a yellow image).

The color space of the *Lab* turned into a color space suitable for machine vision due to its resistance to color variations. The conversion from *RGB* color space to *Lab* was performed in the form of relation (1):

(1) $$L=116\times f\left(\frac{Y}{Y_{n}}\right)-16$$

$$a=500\times \left[f\left(\frac{X}{X_{n}}\right)-f\left(\frac{Y}{Y_{n}}\right)\right]$$

$$b=200\times \left[f\left(\frac{Y}{Y_{n}}\right)-f\left(\frac{Z}{Z_{n}}\right)\right]$$

Where

$$f\left(t\right)=\left\{\begin{array}{c} t^{1/3}\text{if}:t>{\left(\frac{6}{29}\right)}^{3}\\ \frac{1}{3}{\left(\frac{29}{6}\right)}^{2}t+\frac{4}{29},\;\;O.W. \end{array}\right.$$

*Lab* color space is able to describe all colors visible by the human eye and is used as an independent color model. The *Lab* color space is derived from the original XYZ color space (Kubat, 2015). After removing the background of the images, the K‐means method was used to segment images. Moreover, to remove the luminance effect, the component *L* of the images was also deleted and the clustering algorithm was applied only to the components *a* and *b*. The basis of clustering in the algorithm designed was squared Euclidean distance. In this algorithm, the Otsu method was used to carry out an automatic histogram of the threshold of images based on the shape or reduction of gray surface in binary images.

After converting the color space from *RGB* to *Lab*, the colors obtained from the previous step were clustered using the K‐means method. In this algorithm (clustering algorithm), the colors which were similar to each other in the image were placed in a certain number of clusters up to a certain distance, and each of the clusters (3 clusters) was the final result. To use this method, the number of clusters and the spacing of each cluster from the other should be determined. For this purpose, relation (2) was used:

(2) $$\text{ab}=\text{double}\;\;\left(lab\_\text{he}\;\left(:\;\;,\;\;:\;\;,\;\;2:3\right)\right);$$

$$\text{nrows}=\text{size}\;\left(\text{ab},\;\;1\right);$$

$$\text{ncols}=\text{size}\left(\text{ab},\;\;2\right);$$

$$\text{ab}=\text{reshape}\;\;\left(\text{ab},\;\;\text{nrows}*\text{ncols},\;\;2\right);$$

ncolors=3;

$$\left[\text{cluster}\;\;\text{idx}\;\;\text{clustercenter}\right]=\text{kmeans}\;\;\left(\text{ab},\;\;\text{ncolors}{,}^{\prime }{\text{distance}}^{\prime },\;{\;}^{\prime }{\text{sqeuclidean}}^{\prime }{,}^{\prime }{\text{replicates}}^{\prime },\;\;3\right);\;$$

**Figure 6:**
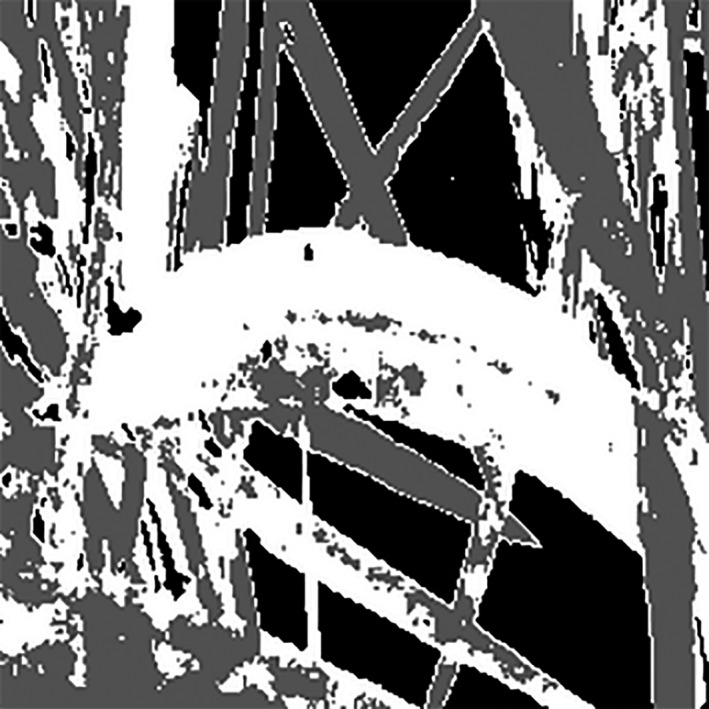
Labeled image in *Lab* space

After specifying the clusters and spacing, an image was labeled by cluster index. Labeling of pixels using the results of the K‐means method for each part of the image shows a cluster index (Figure 6).

**Figure 7:**
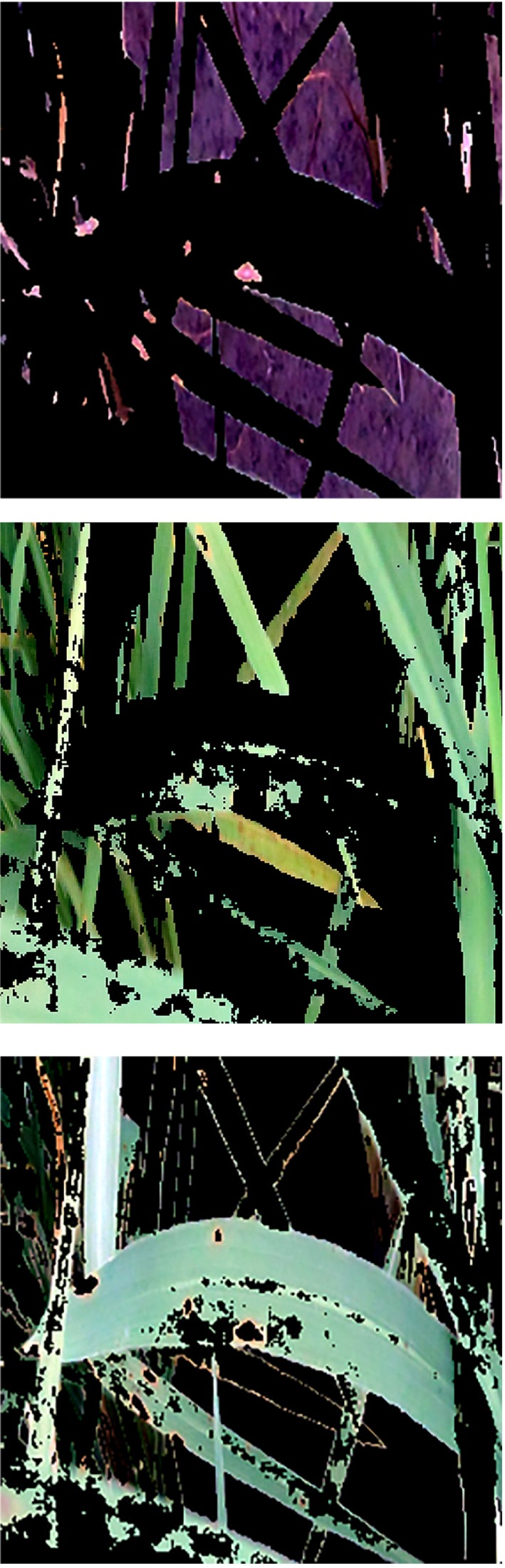
Output images of the algorithm. Cluster 1: Identify the diseased points on the rice leaf and mark those points. Cluster 2: Light green spots on the leaf and stem of the plant and cluster 3: dark green spots on the plant leaf

After executing the image labeling algorithm, the next step is to obtain clustered images. This operation was carried out using equation (3). This stage is the main step in the rice blast disease diagnostic algorithm. The image of the diseased parts, the healthy parts of the plant, and pixels that do need to be processed will be obtained. Each of these images is a kind of cluster of the original image (Figure 7).

(3) $$\text{Segmented}\_\text{images}=\text{cell}\;\;\left(1,\;\;3\right);$$

$$\text{rgb}\_\text{label}=\text{repmat}\;\;(\text{pixel}\_\text{labels},\;\;\left[1\;\;1\;\;3\right];$$

for k=1:ncolors

color=he;

$$\text{color}\;\;\left(\text{rgb}\_\text{label}∼=k\right)=0;$$

$$\text{segmented}\_\text{images}\left\{k\right\}=\text{color};$$

After clustering, classification turn arrives. KNN should be improved for the following reasons:

1. The KNN algorithm is slow since it reviews all the instances each time.
2. The algorithm is vulnerable to dimensionality.
3. The algorithm is sensitive to irrelevant and correlated attributes.
4. A wrong choice of the distance or the value of k degrades the performance.

The KNN algorithm is improved as follows and the classification operation begins:

K‐means algorithm is used to form clusters, and the classification will be based on the centers of this new set of clusters. Thus, classifying a new instance into one of the k clusters instead of comparing it to the initial n instances divides the computation time of the algorithm by k/n. Finally, the distance between a given instance and the center of each cluster is restricted to significant attributes and weighted by their reliability coefficients.

## 3 RESULTS AND DISCUSSION

In this research, with the improvement of KNN algorithm by K‐means, a new, quick and accurate method for diagnosis of rice disease was developed using image processing. The results of this study showed that the dynamic range of gray surfaces should be increased to determine the damaged parts of the rice leaf. Therefore, the numeric data type of images changed from uint 8 type to double data type. Rewar, Singh, Chhipa, Sharma, and Kumari (2017), and Al Bashish, Braik, and Bani‐Ahmad (2011) also used double images to diagnose various plant diseases under controlled, laboratory conditions and their input images were *RGB*.

Moreover, the *Lab* color space enabled us to separate the colors in the image and, using the K‐means clustering algorithm, identify the diseased locations and the spots that have changed color on the rice leaf. The K‐means method is one of the data mining techniques used in machine vision. Clustering is an uncontrolled learning method that does not rely on predefined categories or specific features as objectives, and places instances with the same amount of data together in one group. The objective function in the K‐means clustering was calculated using equation (4):

(4) $$J=\sum_{j=1}^{k}\;\;\sum_{i=1}^{n}{∥x_{i}^{j}-c∥}^{2}$$

where $\left|\right|x_{i}^{j}-c_{j}\left|\right|$ is the measure of the distance between the points and *c_j_* is the center of the *j^th^* cluster. In the designed algorithm, at first *K* points were selected as the centers of the clusters. Then, each sample data were attributed to the cluster whose center had the smallest distance to that data. Finally, the average of the points belonging to each cluster was assigned to one cluster and for each cluster; a new point was calculated as the center.

**Figure 8:**
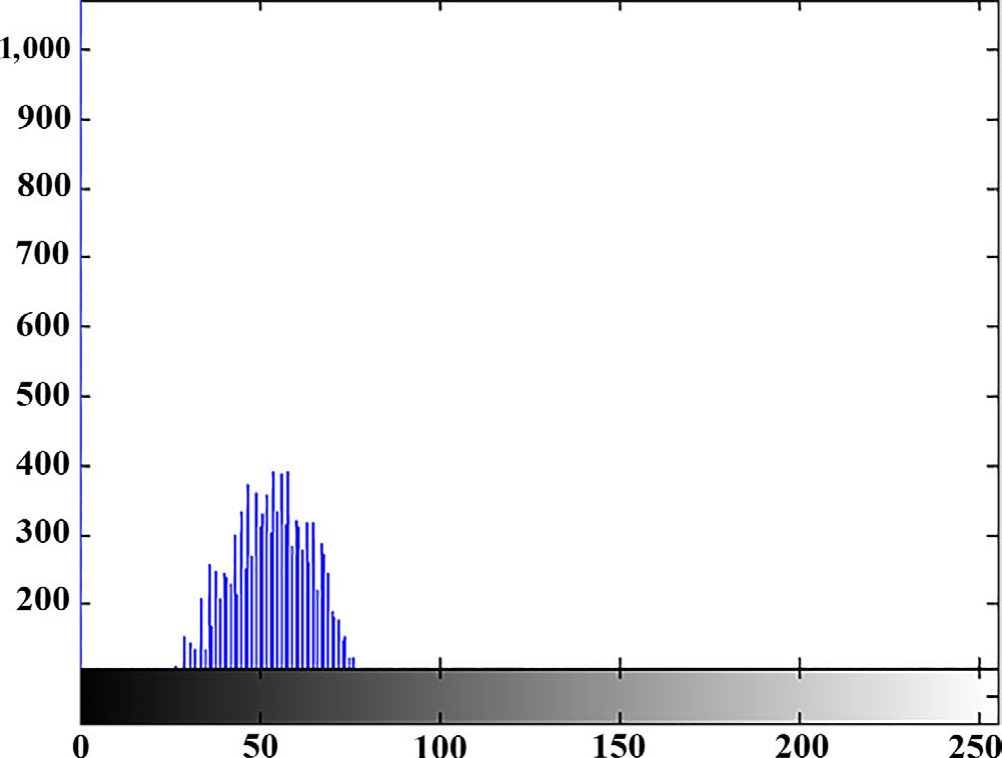
Class I clustering histogram (determining the diseased points on the plant leaf)

**Figure 9:**
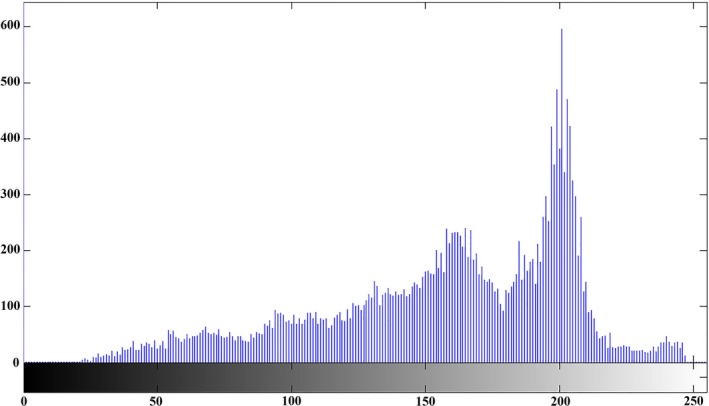
Class II clustering histogram (light green)

**Figure 10:**
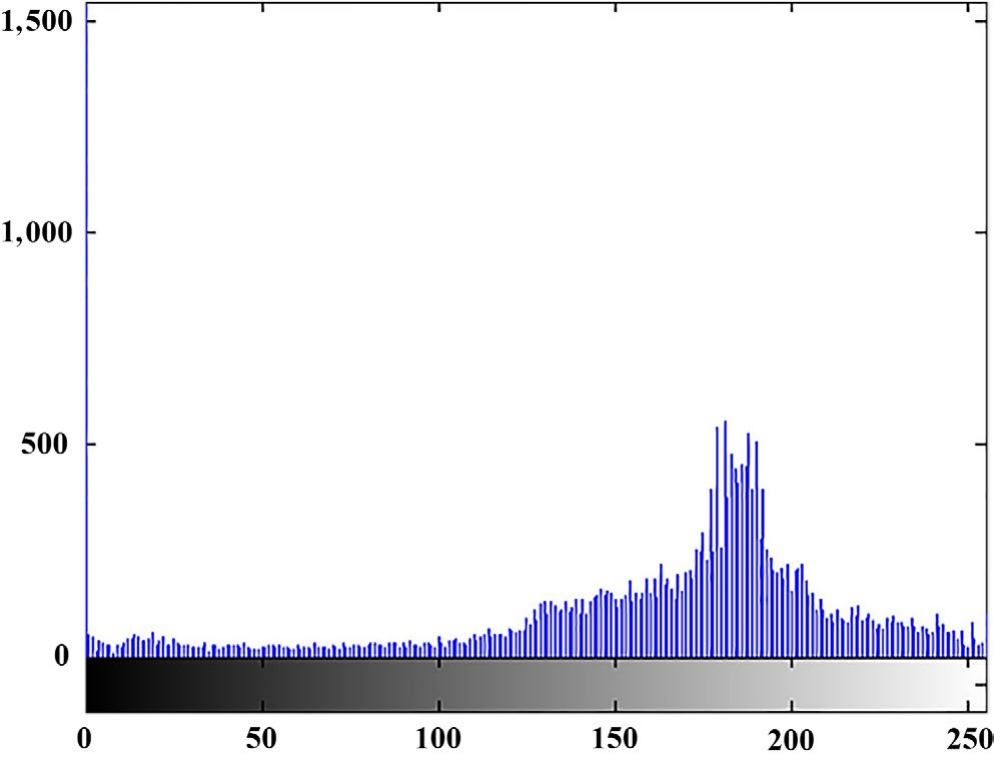
Class III clustering histogram (dark green)

Figures 8, 9, and 10 illustrate the histogram of clustering of images in three classes, respectively. Gray surface area in class 1 clustering in Figure 8 is the threshold value for separating disease spots on rice leaves and other parts of the image. In the Figures 8, 9, and 10 the horizontal axis shows the gray surface level and the vertical axis the number of image pixels.

### 3.1 Accuracy of the designed algorithm

Two criteria of identification number and quality of blast spots were used to assess the ability and accuracy of the proposed algorithm. The results of the confusion matrix for the 500 sample images tested are given in Table 1. The main diameter of the matrix shows the accurate results obtained by the algorithm (Improved KNN algorithm by K‐means).

**Table 1:** The results of testing 500 sample images to determine the number and quality of blast spots

Output	determining the diseased spots	Number of disease spots	pixels other than the diseased spots
Number of diseased spots	20	460	0
The quality of determining the diseased spot	480	40	0
Pixels other than the diseased spots	0	0	500

Three factors of sensitivity, specificity, and accuracy were investigated to determine the efficiency of the designed algorithm.

1. Sensitivity: the number of accurate categories per class/total number of each class.
2. Specificity: ((Number of inaccurate categories of each class/total number of other classes)×100)–100.
3. Total accuracy: total number of classes/total number of accurate categories in all classes.

The amount of each of the above‐mentioned factors is shown in Table 2:

**Table 2:** statistical factors of sensitivity, specificity and total accuracy

Image categories	Statistical factors		
	Sensitivity (%)	Specificity (%)	Total accuracy (%)
Number of diseased spots	92	91.70	
The quality of determining the diseased spot	96	95.65	94
Pixels other than the diseased spots	100	100	

In similar studies, the accuracy of the diagnostic algorithm for various rice diseases was obtained by Venugoban and Ramanan (2014), Zainon (2012), and Qing et al. (2014) to be 90, 92.5, 69.76, and 85.20%, respectively. Compared to other researchers' investigations, this study reported a higher percentage of overall accuracy (94%) in field conditions. In addition, the time spent on the implementation of the algorithm designed to detect rice disease in one of the clusters was 5.894 s.

## 4 CONCLUSION

The results of this study showed that image processing and machine vision have a high potential for determining blast disease in rice plant under field conditions. Moreover, the KNN machine learning method improved by K‐means can be used as an effective diagnostic mechanism for the disease. This method is faster and less expensive than other methods and it is also nondestructive. The observations carried out show that the accuracy of the new method of machine vision is at an acceptable level and the results of this method can be relied upon. Besides that, by carefully managing the fields and by the timely prevention of the rice blast spread in the northern fields of the country, it is possible to avoid the overuse of chemical pesticides resulting in increased production costs, environmental pollution, and reduction in production per hectare.

## CONFLICT OF INTEREST

The authors have declared no conflict of interest.

## ETHICAL APPROVAL

This study does not involve any human or animal testing.

## INFORMED CONSENT

Written informed consent was obtained from all study participants.
